# Recent Progress on Conversion of Lignocellulosic Biomass by MOF-Immobilized Enzyme

**DOI:** 10.3390/polym16071010

**Published:** 2024-04-08

**Authors:** Juan Tao, Shengjie Song, Chen Qu

**Affiliations:** 1 School of Life Science, Jiangxi Science and Technology Normal University, Nanchang 330013, China; taojuan2001@126.com (J.T.); songshengjie@hotmail.com (S.S.); 2 Advanced Institute for Materials Research (AIMR), Tohoku University, Sendai 9808577, Japan

**Keywords:** lignocellulosic biomass, conversion, enzyme, immobilization, metal–organic framework

## Abstract

The enzyme catalysis conversion of lignocellulosic biomass into valuable chemicals and fuels showed a bright outlook for replacing fossil resources. However, the high cost and easy deactivation of free enzymes restrict the conversion process. Immobilization of enzymes in metal–organic frameworks (MOFs) is one of the most promising strategies due to MOF materials’ tunable building units, multiple pore structures, and excellent biocompatibility. Also, MOFs are ideal support materials and could enhance the stability and reusability of enzymes. In this paper, recent progress on the conversion of cellulose, hemicellulose, and lignin by MOF-immobilized enzymes is extensively reviewed. This paper focuses on the immobilized enzyme performances and enzymatic mechanism. Finally, the challenges of the conversion of lignocellulosic biomass by MOF-immobilized enzyme are discussed.

## 1. Introduction

**Figure 1:**
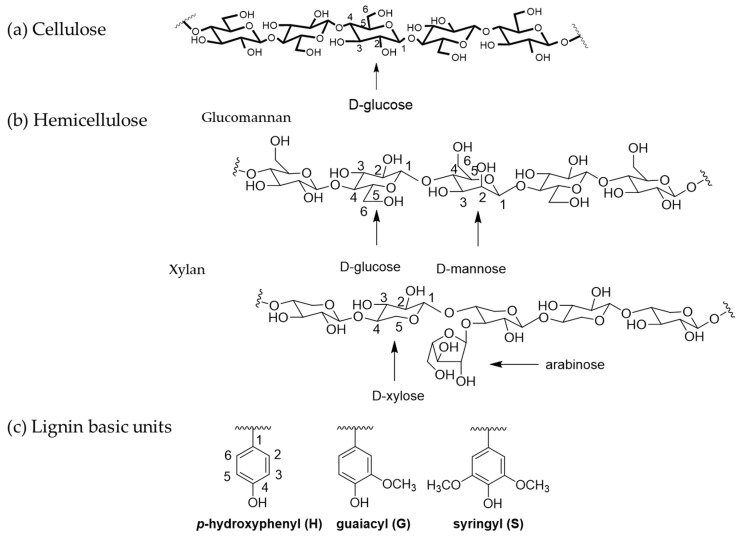
The chemical structures of lignocellulosic biomass main components. (**a**) Cellulose; (**b**) hemicellulose; (**c**) lignin basic units.

Lignocellulosic biomass is a renewable, food non-competitive, and sustainable alternative for replacing fossil resources [1,2]. It is mainly composed of cellulose (40–50%), hemicellulose (20–30%), and lignin (20–30%) (Figure 1). Cellulose is a highly crystallized polymer. The basic unit of cellulose is D-glucosyl units, which are linked with β-(1-4) connections. The chemical structure of hemicellulose is more complicated than cellulose, which includes glucans, xylans, mannans, and xyloglucans, with glucose, xylose, mannose, glucose, galactose, rhamnose, and arabinose as the basic units. Aside from polysaccharides, another main component of lignocellulosic biomass is lignin. Lignin is the second most abundant natural polymer, which has a cross-linked polyphenol structure and is difficult to determine. The main basic units of lignin are *p*-hydroxyphenyl (H), guaiacyl (G), and syringyl (S). Lignocellulosic biomass chemical structures and compositions are varied based on the species of plants [3].

**Figure 2:**
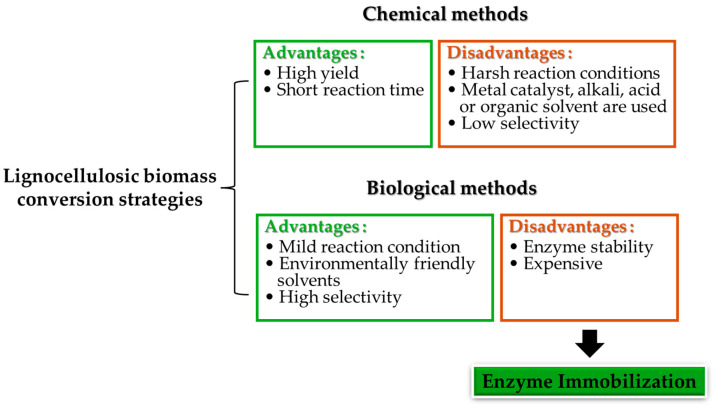
Comparison of the advantages and disadvantages between chemical and biological conversion methods.

However, due to the compact structure, the decomposition of lignocellulosic biomass into available small compounds needs harsh conditions. Different strategies have been developed for the conversion of lignocellulosic biomass components into chemicals, materials, and energies [4]. Chemical conversion methods and biological conversion methods are the two main routes for realizing this objective. Both conversion methods have their advantages and disadvantages (Figure 2). For chemical conversion methods, the conversion reaction normally needs harsh reaction conditions such as high reaction temperature and/or high reaction pressure. Also, alkali, acid, metal catalysts, and hydrogen are sometimes needed for the conversion process [5]. Furthermore, during the chemical reaction process, various kinds of products can be obtained, so the separation and purification of the complicated products are also a challenge for the valorization of lignocellulosic biomass [6].

Biological conversion methods have attracted the attention of researchers due to their mild reaction conditions and high selectivity [7]. On the other hand, because of the complicated structure of lignocellulosic biomass, several specialized enzymes are required for lignocellulosic biomass, which are mainly classified into cellulases, hemicellulases, and ligninases [8,9,10]. The free enzyme is extremely fragile and is readily inactivated by heat and many disinfectants, including organic solvents, strong acids or bases, and some metal ions. There are several technologies that have been proposed to improve the stability of enzymes, such as immobilization, protein engineering, and chemical modification [11,12,13]. Among those technologies, immobilization is a feasible and effective strategy, which is achieved by fixing the enzyme to a suitable support through physical adsorption, entrapment, chemical cross-linking, or covalent binding [14,15]. An ideal support for this objective should have good associativity, stability, physical strength, and reproducibility and should be chemically inert. Many conventional support materials, such as chitosan, resin, silicon, etc., were studied for this aim [16]. However, poor mechanical and chemical stability, non-uniform pore distribution, poor biocompatibility, and/or lack of functional groups limited the utilization of the above materials for enzyme immobilization. Hence, developing a new carrier matrix to meet all of these needs is essential for enzyme immobilization.

A metal–organic framework (MOF) is a type of organic–inorganic hybrid crystalline porous material with large surface area, adjustable pore sizes, and good biocompatibility, and it has been widely applied in gas adsorption and separation, drug delivery, catalysis, and other fields [17,18]. In light of the active sites and ligand functionalization, MOFs and their derivatives could be used to catalyze lignocellulosic biomass to various products through hydrolysis, dehydration, isomerization, oxidation, etc. Recently, MOFs attracted considerable attention as potential materials for enzyme immobilization [19,20,21,22]. Various organic linkers and metal nodes are available to tailor MOFs with tunable properties so that MOFs can be adapted for the fixing and protection of enzymes [23]. Herein, this paper reviewed recent progress on the conversion of lignocellulosic biomass by MOF-immobilized enzymes, summarizing the bioconversion of cellulose, hemicellulose, and lignin, respectively. The prospect of the conversion of lignocellulosic biomass by MOF-immobilized enzymes is discussed. In contrast to other review papers using MOFs or MOF derivatives as chemical catalysts, this review aims to provide constructive suggestions for a better understanding of the utilization of immobilized enzymes in MOFs for lignocellulosic biomass conversion.

## 2. Conversion of Cellulose by MOF-Immobilized Enzyme

Cellulose is a linear polysaccharide consisting of 3000 or more glucose units linked by β-1,4-glycosidic bonds. There are multiple intramolecular and intermolecular hydrogen bonds in natural cellulose, resulting in poor accessibility for enzymes [24]. Therefore, most of the literature has applied microcrystalline cellulose (MCC) as the substrate, which is a kind of freely flowing powder particle with a degree of polymerization (DP) of 15–375 [25].

**Figure 3:**
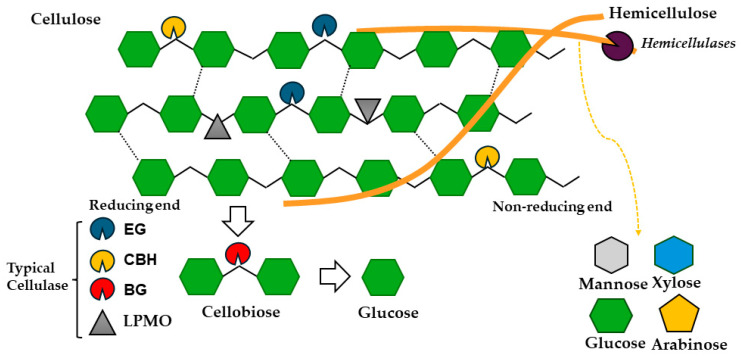
Schematic diagram of polysaccharide degradation by cellulase (endo-β-1,4-glucanase (EG), exo-β-1,4-glucanase (CBH), and β-glucosidase (BG)), lytic polysaccharide monooxygenase (LPMO), and hemicellulases.

The most widely used enzyme for the bioconversion of cellulose is cellulase. Cellulase is a multi-component enzyme, including endo-β-1,4-glucanase (EG), exo-β-1,4-glucanase or cellobiohydrolase (CBH), and β-glucosidase (BG) [26]. The generally accepted enzymatic mechanism is that EG arbitrarily breaks the interior β-1,4-glycosidic bond, CBH breaks the β-1,4-glycosidic bond from the reducing or non-reducing end to produce cellobiose, and BG resolves the cellobiose to glucose [27]. Lytic polysaccharide monooxygenase (LPMO) is a newly discovered oxidase and is classified as an auxiliary active (AA) enzyme. It could break glycosidic bonds to generate more glycoside binding sites, thus accelerating the enzymatic reaction process (Figure 3). However, the exact catalytic mechanism of LPMO has not been well understood, and it opens a new way for enzymatic degradation of the cellulose.

**Figure 4:**
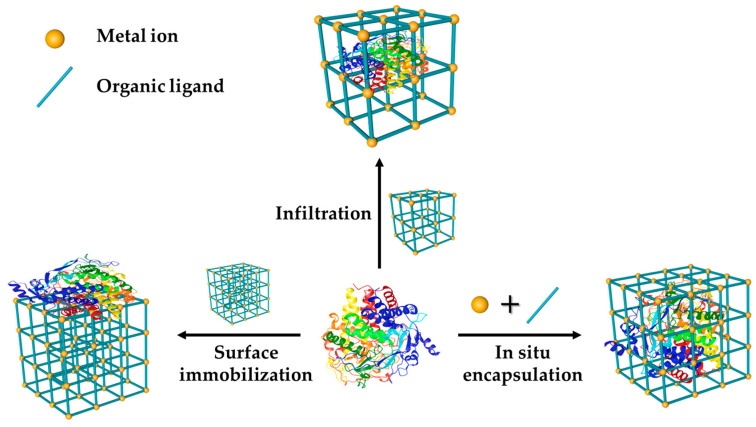
Three methods of cellulase immobilization by MOF.

Because of the sensitivity and relatively high price of enzymes, immobilization of cellulase in MOFs is a facile approach to improve its stability and recyclability [28]. There are three main immobilization ways, which include surface immobilization, in situ encapsulation, and infiltration (Figure 4). For the surface immobilization method, cellulase is fixed in MOF through physical adsorption or chemical binding. It is the most frequently used method due to its easy operation and minimal effect on cellulase activity. For microporous MOF materials, cellulase is usually immobilized on external surfaces, and the leaching of cellulase easily occurs during the reaction process, which in turn results in a loss of activity upon reuse. To strengthen the linkage, MOF is modified by functional groups, such as NH_2_, and then forms covalent bonds with cellulase via glutaraldehyde cross-linking [29]. The disadvantage of this method is that it is difficult to use MOF’s rich pore structure to realize the surface immobilization method. For the in situ encapsulation method, cellulase is added during the formation of MOF and then encapsulated in the framework of MOF through coprecipitation [30]. The preparation of MOF must be conducted under mild conditions. Otherwise, cellulase will be easily inactivated during immobilization. The optimal operation conditions of cellulase are at pH 5.0 and 50 °C [31]. Therefore, researchers have focused on developing new MOFs prepared in slightly acidic solution at lower temperatures. This method enhances the binding between cellulase and MOF, and the framework has great protection against cellulase. However, the activity of the in situ encapsulated enzyme was questioned by some researchers. The third immobilization process is infiltration, and cellulase diffuses into the pores or cages of MOFs [32]. The short axis and long axis of cellulase are 3.8 nm and 17.6 nm [33]. Only MOF with a considerable pore size could immobilize cellulase in pores without leaching. So far, there are a few macro- or mesoporous MOFs reported owing to the conventional technical restriction [34].

Table 1 summarizes the progress of the conversion of cellulose by MOF-immobilized enzymes. Surface immobilization and in situ encapsulation are the main immobilization methods. The NH_2_-functionalized MOF exhibits higher enzyme loading due to the extra anchor sites of NH_2_ groups [35]. With increasing enzyme concentration, the loading capacity of immobilized cellulose increases. But the specific activity first increases and then decreases, which may be attributed to the tight and compact loading of the proteins that result in serious steric hindrance. Hence, both the loading capacity and specific activity should be considered to optimize the dosage of enzyme and MOF. Qi et al. reported that magnetic MOF could be easily recovered from the solution, but magnetic nanoparticles (Fe_3_O_4_, etc.) reduced the number of available binding sites of the enzyme [29]. The immobilized cellulase has higher stability than free cellulase, which could keep a high activity at a wide range of pH values and temperatures. All immobilized enzymes shown in Table 1 can be reused many times, and for some of them, the residual activity is up to 90%. The storage stability is also improved (data not listed). For example, the activity of cellulase@UiO-66-NH_2_ could retain 65% after 30 days of storage [35]. The results have suggested the promising future of cellulase–MOF composites for practical applications.

## 3. Conversion of Hemicellulose by MOF-Immobilized Enzyme

Hemicellulose is a hetero-polymerized polysaccharide composed of two or more monosaccharides with side chains and branched chains [40]. It has a very low DP (100–200) compared with cellulose. The monosaccharides mainly include hexoses (D-glucose, D-mannose, and D-galactose) and pentoses (D-xylose, L-arabinose, and D-arabinose). The composition of hemicellulose varies among different plant species, which could be divided into glucomannans, xylan, galactoglucomannans, xyloglucans, etc. [41]. D-pyranyl glucose and pyranyl mannose are linked by a 1,4-β form bond to form the backbone of glucomannans. Xylan is linked by xylose via a β-(1→4) glycosidic bond, and substitution of glucuronic acid, 4-O-methyl-glucuronic acid, or arabinose may occur in C2 and C3.

In view of the complex constituents and linkages of hemicellulose, it requires the combination of multiple specific enzymes to degrade hemicellulose to oligosaccharides, disaccharides, and monosaccharides [42]. Each hemicellulase is a composite enzyme. For example, xylanase includes β-1,4-endoxylanase, β-xylosidase, α-L-arabinosidase, α-D-glucuronidase, acetyxylanase, and phenolylesterase. Similar to cellulase, each component has a distinct action site that ultimately degrades hemicellulose into monosaccharides [43]. Although it requires various enzymes, the enzymatic reaction of hemicellulose is relatively easy for its lower DP and incompact structure [44].

Few investigations about the bioconversion of hemicellulose have been reported to be attributed to its complex compositions. Most of the reported studies use isolated xylan as model hemicellulose for the study. It is because on the one hand, xylan is the main constituent of hemicellulose. Meanwhile, xylooligosaccharides (XOSs) and xylose, as degradation products of xylan, have a wide market perspective. XOSs could promote calcium absorption, reduce cholesterol levels and the risk of colorectal cancer, and have antioxidant and antibacterial activity [45]. Xylose is a precursor for the production of important chemicals such as xylitol, ethanol, and lactic acid. Kaushal et al. reported an efficient bioconversion method to obtain xylotetrose (X5) and xylopentose (X6) by both free and Cu-BTG-immobilized xylanase. The results suggested that the immobilization of the xylanase enzyme helps the enzyme selectively produce XOS from the extracted xylan in large quantities, with the conversion yield being at 11.8% X4 and 64.2% X5 [46]. Gui et al. developed an MOF-immobilized enzyme Fe_3_O_4_@PDA@MOF-Xy, which has significant advancement loading capacity (80.67 mg/g) in protein and exhibits remarkable tolerance to acidic and alkaline conditions. Furthermore, the yield of xylooligosaccharides from corn cob xylan was 1.15 times higher than that of the free enzyme system [47]. Table 2 summarizes conversion of xylan by enzyme immobilized in MOFs. Compared with free enzyme, the immobilized enzyme not only has superior stability and reusability but also has higher catalytic activity [47,48]. The reasons may be the change in microenvironment and partition effects after immobilization, which effect conformational, steric, and mass transfer processes and then effect the catalytic activity.

## 4. Conversion of Lignin by MOF-Immobilized Enzyme

**Figure 5:**
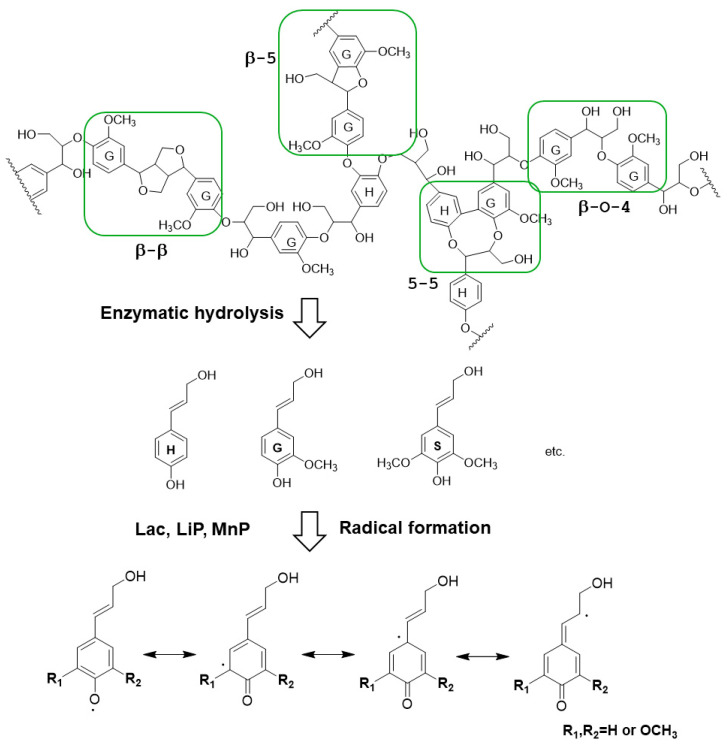
A fragment of the lignin chemical structure and its schematic diagram of degradation by laccase (Lac), lignin peroxidases (LiP), and manganese peroxidase (MnP).

Lignin is a three-dimensional net polymer cross-linked by phenyl propane monomer through ether bonds and carbon–carbon bonds (Figure 5). The most common linkages are β-O-4 (45–50%), 5-5 (18–25%), β-5 (9–12%), β-1 (7–10%), α-O-4 (6–8%), and 4-O-5 bonds (4–8%) [51]. Lignin contains a variety of active functional groups, such as methoxy, hydroxyl, epoxy, carboxyl, and the like, which provide lignin with an additional functional property [52]. Lignin degradation is very difficult. The complete degradation of lignin in nature is the result of the combination of fungi, bacteria, and the corresponding microbial communities [53]. Due to its complex structure and high molecular weight, the natural degradation rate of lignin is extremely slow. Researchers have focused on exploiting new microorganisms for effectively bioconverting lignin. In the presence of microorganisms or enzymes, the connection bonds between the monomers are broken and decomposed into low molecular compounds. The most studied degrading enzymes of lignin are laccase (Lac), lignin peroxidases (LiP), and manganese peroxidase (MnP) [54].

Lac is thought to be the starting enzyme and is an oxidant for degrading phenolic lignin without requiring H_2_O_2_ [55]. The optimum pH and temperature of Lac are 2–10 and 40–65 °C. Lac has four copper atoms and is divided into three types (Type Ⅰ Cu^2+^, Type Ⅱ Cu^2+^, and Type Ⅲ Cu^2+^). The degradation mechanism of Lac needs four single-electron transfers. Lac removes four electrons from the hydroxyl groups of phenolic compounds to transfer to Type Ⅰ Cu^2+^ under the action of oxygen in the environment. This process also forms four phenoxy radical intermediates. Then, electrons are transferred to Type Ⅱ Cu^2+^ through a Cys–His pathway, where it binds oxygen and is reduced to water. Meanwhile, the four phenoxy radicals are unstable and undergo non-enzymatic reactions, resulting in further cleavage of lignin [56]. LiP plays a key role in lignin biological degradation. LiP could oxidize phenolic or non-phenolic aromatic ring multimers and break C_α_-C_β_ bonds in side chains of lignin into monomers. LiP has an optimum pH of 2–5 and an optimum temperature of 35–55 °C. The reaction mechanism of LiP is that LiP is firstly oxidized by H_2_O_2_ to the unstable intermediate LiP Ⅰ, and then LiP Ⅰ accepts a single electron extracted from the substrate to form LiP Ⅱ, which reduces Fe^4+^ to Fe^3+^ by transferring an electron from another substrate to restore the initial state. The generated two cationic radicals could attack other chemical bonds of lignin through subsequent non-enzymatic reactions, thus causing polymerization of lignin [57]. MnP is another key enzyme for lignin degradation and only oxidizes phenolic lignin in the presence of H_2_O_2_. The optimal pH and temperature of MnP are 4–7 and 40–60 °C. Mn^2+^ of MnP is oxidized to Mn^3+^, and Mn^3+^ in turn oxidizes phenol to phenoxy residues, which undergo a series of reactions to crack the lignin structure [58].

However, the degradation rate of lignin is low when using the above enzymes individually, and Lac has the highest lignin percentage degradation, with a degradation of only 11.73%. When Lac, LiP, and MnP are used synergistically, the lignin degradation rate improves up to 25.79% [59]. This is because Lac can oxidize a wide range of substrates and therefore break different linkages of lignin. Meanwhile, Lac has the role of depolymerizing and polymerizing lignin, and a high lignin degradation rate is achieved in the presence of LiP or MiP, which can prevent the polymerization of lignin [60]. Lac also plays a positive role in promoting the enzymatic action of LiP and MnP. The key degradation pathways by Lac, LiP, and MnP are shown in Figure 5. Lignin or monomers as model lignin are oxidized under enzymatic catalysis to form radicals, which could attack other chemical bonds of lignin for further depolymerization. Meanwhile, the enzymes could also break the unstable aromatic ring to form aliphatic compounds, such as acids, alcohols, ketones, esters, etc.

So far, little research about the conversion of lignin by MOF-immobilized enzyme have been reported. The related literature mainly focuses on phenolic compounds degraded by Lac immobilization in MOFs [61]. As shown in Table 3, surface immobilization and in situ encapsulation are also the main immobilization methods for Lac because of its larger size (6.5 nm × 5.5 nm × 4.5 nm) [62]. After immobilization, the stability of Lac is remarkably improved, and the immobilized Lac has a wider range of pH and temperature than free Lac. The reusability and storage stability of immobilized Lac are also enhanced. The kinetic parameters are determined to study the effect of immobilization on the rates of enzyme-catalyzed reactions. In general, the V_max_ values of immobilized Lac are lower than that of the free Lac. The V_max_ value of immobilized Lac was 86.7% that of free Lac [63]. The decrease in V_max_ might be attributed to the mass transport constraint [64,65]. The K_m_ value of Lac mostly increases, indicating the weaker binding ability of immobilized Lac to the substrate [66,67]. The reasons may be the structure alteration and loss of enzyme flexibility. However, the K_m_ value could also decrease after immobilization, and the alteration of enzyme structure may generate more active sites. The K_m_ values of the free and immobilized Lac are 436.8 μM and 306.1 μM, and the immobilized Lac has a stronger affinity [68]. Significantly, the residual catalytic activity of immobilized Lac can remain high, being up to 63.42% and 46.17% for OPEO and NPEO after four cycles [69]. Lac/Co-MOF can remove 78% RB171 and 61% RB198 at the fifth cycle [70].

Recently, MOF enzyme mimics, with similar catalytic activities to their natural counterparts, are ideal alternatives used in biomass conversion for their tunable structures, high stability, and low cost [72,73]. Inspired by the multicopper active site of Lac, MOFs contain multiple metal ions and could be made to mimic Lac. Liang et al. firstly synthesized a functional Lac mimic based on guanosine monophosphate (GMP)-coordinated copper, named Cu/GMP. It is an amorphous MOF with a higher V_max_ and similar K_m_ to Lac [74]. Shams et al. designed a Lac mimic Cu/H_3_BTC MOF that possessed fundamental activities for the oxidation of phenolic compounds. In addition, the degradation of azo dye AB-10B by Cu/H_3_BTC was up to 60% after ten cycles [75]. Wang et al. reported an amorphous MOF-based nanozyme (CA-Cu) with both laccase- and catecholase-like activity. It has higher degradation efficiency for environmental phenolic pollutants [76]. Liang et al. designed Ce-UiO-66 and Ce-MOF-808, where the internal cerium redox (Ce^4+^/Ce^3+^) reactivity could mimic the active site and catalytic function of Lac. Ce-UiO-66 and Ce-MOF-808 had superior stability and recyclability toward the oxidation of phenolic compounds [77]. Yang et al. found that Cu/GMP shows superior Lac-like activity for the C-O bond cleavage of lignin, which could degrade organosolv lignin (OL) into oligomers with low molecular weights in a high yield (81.7 wt%) [78].

## 5. Summary and Prospective

In this review, recent progress on the conversion of cellulose, hemicellulose, and lignin by MOF-immobilized enzyme was summarized, in which MOF plays a key role in enhancing the stability and reusability of enzymes. Nevertheless, the bioconversion of lignocellulosic biomass is limited to its compact structure and complex composition. Most of the articles cited above summarized the use of isolated components from lignocellulose biomass or simple model compounds, such as MCC, xylan, etc., as substrates for free and immobilized enzymes. Given the immobilization technical constraints, the immobilized enzyme in MOFs suffers from denaturation, leaching, poor biocompatibility, and so on.

Consequently, further work on the conversion of lignocellulosic biomass has focused on the following aspects: (1) New pretreatment strategies should be developed to improve the accessibility of enzymes. Pretreatments are vital to lignocellulosic biomass to modify its structure and chemical composition. Developing effective solvents, such as ionic liquids and *p*-TsOH aqueous solution, is a promising strategy to separate three main constituents from lignocellulosic biomass and transfer its compact structure to an uncompact amorphous state at mild conditions [79,80]; (2) New meso- or macro-porous MOFs should be designed. High transfer resistance is a critical shortcoming for micropore MOF, which will weaken the enzymatic efficiency. Furthermore, a variety of enzymes are required to act synergistically to degrade lignocellulosic biomass. It requires MOFs that possess larger pore sizes to immobilize two or more enzymes; (3) Biocompatible MOFs will become a focus of research. Many decomposable compounds of lignocellulosic biomass will be applied in food, medicine, cosmetics, and dresses, so MOFs with good biocompatibility are ideal enzymes supports [81]; (4) MOFs as enzyme mimetics will gain more and more attentions in the bioconversion of lignocellulosic biomass due to its high stability and low cost [82]; (5) MOFs could be used to catalyze the lignocellulosic biomass to value-added products. When MOFs were used as enzyme supports, their catalytic activities were rarely discussed. The synergistic effects of enzymes and MOFs need further research to fully utilize the potential of nanomaterials in the bioconversion of lignocellulosic biomass. Overall, conversion of lignocellulosic biomass by MOF-immobilized enzyme will be a hot topic in future.

## Figures and Tables

**Table 1:** MOF-immobilized enzymes for cellulose conversion.

MOF	Immobilization Method	Maximum Loading (mg/g)	Optimal Reaction Conditions	Recycling Times	Residual Activity (%)	Reference
Fe_3_O_4_@UIO-66-NH_2_	Surface immobilization	126.2	pH 5.0, 50 °C	5	70	[29]
UiO-66-NH_2_	Surface immobilization	350	pH 5.0, 55 °C	10	72	[35]
Cu-MOF	In situ encapsulation	162.95	pH 5.0, 60 °C	10	90	[36] ^1^
ZIF-8	Surface immobilization	176.16	pH 5.0, 60 °C	5	56 ^2^	[37]
Zn-mIm	In situ encapsulation	350	pH 4.8, 50 °C	4	77	[30]
MOF(PABA)	In situ encapsulation	176.9	pH 4.8, 110 °C	5	86 ^3^	[38] ^1^
MOF-Fe	In situ encapsulation	224.8	pH 4.8, 120 °C	5	30 ^3^	[39] ^1^

^1^ The enzyme is β-glucosidase in the references. ^2^ The reusability of immobilized cellulase is utilized in the process of saccharification with 25% [Emim]DEP. ^3^ The reusability of immobilized β-glucosidase is utilized in the hydrolysis of cellobiose in ionic liquid.

**Table 2:** MOF-immobilized enzyme for xylan conversion.

Enzyme	MOF	Immobilization Method	Recycling Times	Residual Activity (%)	Product Conversion Efficiency (%)	Reference
Xylanase	Cu-BTC	Surface immobilization	-	-	87.4 (XOS)	[46]
GH 11 endo-β-1,4-xylanase	Fe_3_O_4_@PDA@MOF	Surface immobilization	10	60	23 (XOS)	[47]
Xylanase	ZIF-67	In situ encapsulation	8	70	94.73 (Reducing sugar)	[48]
	Mn/ZIF-67		8	70	84.13 (Reducing sugar)	
Xylanase	MOFCu-BTC	Surface immobilization	21	61	57.97 (Reducing sugar)	[49]
β-Xylosidase/endoxylanase	UiO-66-NH_2_	Surface immobilization	5	70	30 (Reducing sugar)	[50]

**Table 3:** MOF-immobilized enzyme for lignin conversion.

MOF	Immobilization Method	Maximum Loading (mg/g)	Optimal Reaction Conditions	Recycling Times	Residual Activity (%)	Substrate Conversion Efficiency (%)	Reference
Zr-MOF, MMU	Surface immobilization	221.83	pH 4.0 40 °C	10	50	-	[63]
Cu-MOF	Surface immobilization	502	pH 4.0 50 °C	7	50	-	[64]
NH_2_-MIL-53(Al)	In situ encapsulation	625	pH 3.0 30 °C	10	63	-	[65]
meso-MIL-53(Al)	Surface immobilization	218	pH 5.0 45 °C	8	60	99.24 (Triclosan)	[66]
Fe_3_O_4_-NH_2_@MIL-101(Cr)	Surface immobilization	69	pH 4.0 65 °C	3	40	85 (2,4-Dichlorophenol)	[67]
Fe_3_O_4_@ZIF-8	Surface immobilization	-	pH 7.0 80 °C	7	-	100 (Indigo carmine)	[68]
Fe_3_O_4_-NH_2_@MIL-100(Fe)	Surface immobilization	61.60 ± 2.92	pH 5.0 50 °C	4	-	100 (OPEO) ^1^ 98.16 (NPEO)	[69]
Co-MOF	Surface immobilization	-	pH 4.5 50 °C	12	56.5	88 (RB171) 77 (RB198)	[70]
Cu-MOF		-	pH 5.0 50 °C	12	55.8	89 (RB171) 39 (RB198)	
Cu_2_O@MOF	Surface immobilization	148	pH 4.0 55 °C	-	-	82.5 (2,4-Dichlorophenol)	[71]

^1^ OPEO: Octylphenol polyethoxylated. NPEO: Nonylphenol polyethoxylated.

## Data Availability

The data presented in this study are available in the article.
